# Dicer-2 Processes Diverse Viral RNA Species

**DOI:** 10.1371/journal.pone.0055458

**Published:** 2013-02-12

**Authors:** Leah R. Sabin, Qi Zheng, Pramod Thekkat, Jamie Yang, Gregory J. Hannon, Brian D. Gregory, Matthew Tudor, Sara Cherry

**Affiliations:** 1 Department of Microbiology, University of Pennsylvania, Philadelphia, Pennsylvania, United States of America; 2 Penn Genome Frontiers Institute, University of Pennsylvania, Philadelphia, Pennsylvania, United States of America; 3 Department of Biology, University of Pennsylvania, Philadelphia, Pennsylvania, United States of America; 4 Department of Cell and Developmental Biology, University of Pennsylvania, Philadelphia, Pennsylvania, United States of America; 5 Watson School of Biological Sciences, Howard Hughes Medical Institute, Cold Spring Harbor Laboratory, Cold Spring Harbor, New York, United States of America; French National Center for Scientific Research - Institut de biologie moléculaire et cellulaire, France

## Abstract

RNA silencing pathways play critical roles in gene regulation, virus infection, and transposon control. RNA interference (RNAi) is mediated by small interfering RNAs (siRNAs), which are liberated from double-stranded (ds)RNA precursors by Dicer and guide the RNA-induced silencing complex (RISC) to targets. Although principles governing small RNA sorting into RISC have been uncovered, the spectrum of RNA species that can be targeted by Dicer proteins, particularly the viral RNAs present during an infection, are poorly understood. Dicer-2 potently restricts viral infection in insects by generating virus-derived siRNAs from viral RNA. To better characterize the substrates of Dicer-2, we examined the virus-derived siRNAs produced during the *Drosophila* antiviral RNAi response to four different viruses using high-throughput sequencing. We found that each virus was uniquely targeted by the RNAi pathway; dicing substrates included dsRNA replication intermediates and intramolecular RNA stem loops. For instance, a putative intergenic RNA hairpin encoded by Rift Valley Fever virus generates abundant small RNAs in both *Drosophila* and mosquito cells, while repetitive sequences within the genomic termini of Vaccinia virus, which give rise to abundant small RNAs in *Drosophila*, were found to be transcribed in both insect and mammalian cells. Moreover, we provide evidence that the RNA species targeted by Dicer-2 can be modulated by the presence of a viral suppressor of RNAi. This study uncovered several novel, heavily targeted features within viral genomes, offering insight into viral replication, viral immune evasion strategies, and the mechanism of antiviral RNAi.

## Introduction

RNA silencing pathways are critical regulators of gene expression in organisms ranging from yeast to humans. RNA silencing is initiated by the action of RNase III enzymes, which target RNAs with dsRNA character to generate small RNA duplexes [1]. Following biogenesis, small RNA sorting directs the selection of one strand of the duplex to be retained and stabilized by an Argonaute family member in RISC [2]. The RNA silencing pathways of *Drosophila melanogaster* have been extensively characterized and allow for detailed dissection of the mechanisms governing RNAi-mediated gene silencing. The siRNA silencing pathway is responsible for controlling the expression of both endogenous and exogenous targets in somatic cells. siRNA biogenesis is carried out by Dicer-2 (Dcr-2), which generates predominantly 21 nucleotide (nt) products from several types of precursors [3]. Endogenous siRNAs (esiRNAs) are generated from long hairpins, retrotransposons or convergent transcription units and regulate endogenous gene expression, while exogenous siRNAs (exo-siRNAs) are processed from long dsRNAs, a process commonly manipulated in molecular biology to achieve targeted gene silencing [1]. In addition, virus-derived siRNAs (vsiRNAs) are cleaved from viral RNAs produced during infection [4]. The miRNA pathway generates mature miRNAs from stem-loop structures within pri-miRNA transcripts through a series of RNase III cleavage events; Drosha first liberates the pre-miRNA from the primary transcript, then Dicer-1 (Dcr-1) generates the mature product, generally 22–23 nt in length [1]. The piRNA pathway, active predominantly in the germline, targets transposon-derived RNAs to produce 24–29 nt piRNAs, although the enzymatic processing and secondary structure of piRNA pathway substrates is poorly understood [1], [2].

In *Drosophila*, siRNAs are preferentially loaded into Argonaute 2 (Ago2), while miRNAs predominantly bind Argonaute 1 (Ago1) [2], [3]. piRNAs are bound by piwi, aubergine and Ago3 complexes [1]. Several properties contribute to small RNA sorting into Argonaute complexes in *Drosophila*, including the thermodynamic characteristics of the small RNA duplex, specific terminal nucleotide sequences, and the extent of central mismatches or pairing within the duplex [2], [4]. However, the properties governing Dcr-2-mediated siRNA biogenesis and dsRNA processing have not been extensively characterized.

Many invertebrates such as plants, insects and *C. elegans* utilize the activity of RNA silencing pathways to degrade viral RNAs and thus limit virus infection [4], [5]. Antiviral RNAi restricts viral infection at two steps, first through viral RNA cleavage by Dicer enzymes to generate vsiRNAs, and secondly through the targeting of viral RNAs by RISC. Numerous medically important viruses are transmitted to humans by insects and are therefore termed arboviruses (arthropod-borne viruses), and studies of natural mosquito vectors have demonstrated that the RNAi pathway is a critical component of the insect antiviral response toward these RNA viruses [6]–[11]. Therefore, uncovering the mechanism of antiviral RNAi is essential to understand the pathogenesis of arboviral infections in insect hosts. *Drosophila* has provided an excellent model for the study of antiviral RNAi, due in part to the fact that the organism is readily infected by a diverse panel of viruses and that its small RNA silencing pathways are well-characterized [1], [12].

Since the *Drosophila* siRNA pathway is both antiviral and exquisitely poised to recognize dsRNA, the prevailing view is that viral dsRNAs generated during replication of RNA viruses are the target of the antiviral silencing system [4], [6] and are thought to represent the Pathogen Associated Molecular Patterns or PAMPs for this innate defense program. Thus, Dcr-2 is the Pattern Recognition Receptor or PRR responsible for targeting the viral PAMPs to induce the antiviral program. In order to better understand the mechanisms by which RNA silencing is antiviral in insects, and to uncover the viral RNA PAMPs that are specifically targeted by the PRR Dcr-2, we used high-throughput small RNA sequencing to characterize the vsiRNAs produced during infection of *Drosophila* cells. We sequenced the small RNAs generated from three RNA viruses with different coding strategies, and one DNA virus. The viruses include the rhabdovirus Vesicular Stomatitis virus (VSV), the human arbovirus Rift Valley Fever virus (RVFV), the natural *Drosophila* picorna-like virus Drosophila C virus (DCV), and the dsDNA poxvirus Vaccinia virus (VACV). By studying the antiviral targeting of this diverse set of viruses, we found common features of viral RNA that are preferentially targeted by the cellular machinery, along with novel, heavily targeted viral RNA structures that have not previously been characterized in either RNA or DNA viruses.

## Results

### The Antiviral RNAi Pathway Targets VSV dsRNA Replication Intermediates

**Figure 1:**
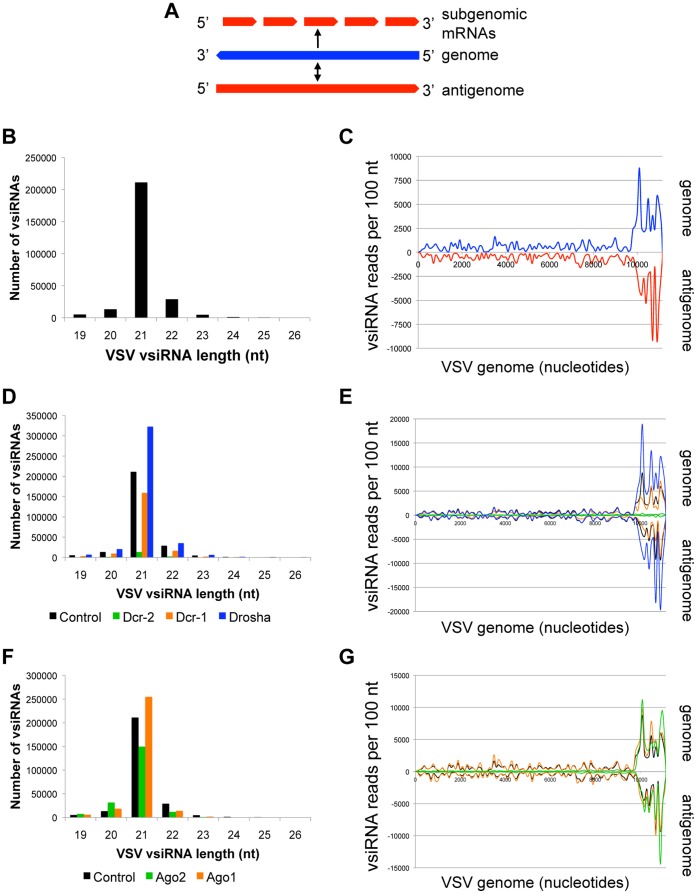
VSV vsiRNAs are concentrated at the 5′ genomic terminus. (A) RNA species produced during VSV infection. (−) strand genome is depicted in blue, (+) strand antigenome and mRNAs in red. (B) VSV vsiRNA size distribution (control library). (C) Distribution of 21 nt VSV vsiRNAs across the viral genome. vsiRNAs mapping to genomic strand are depicted in blue, antigenomic strand in red. (D) VSV vsiRNA size distribution between libraries depleted of RNase III enzymes. (E) Effect of RNase III enzyme depletion on 21 nt VSV vsiRNAs. vsiRNAs from control (black), Dcr-1 (orange), Dcr-2 (green) and Drosha (blue) depleted cells are compared. (F) VSV vsiRNA size distribution between libraries depleted of Argonaute proteins. (G) Effect of Argonaute depletion on 21 nt VSV vsiRNAs. vsiRNAs from control (black), Ago1 (orange), and Ago2 (green) depleted cells are compared. See also Figures S1, S2.

VSV is a rhabdovirus that is naturally transmitted to vertebrates by biting flies, readily infects *Drosophila*, and serves as a model for medically important pathogens such as measles, rabies, and Ebola virus [13]. The virus contains an 11 kb (−) sense RNA genome that is replicated using a (+) sense antigenome as a template (Figure 1A). Viral proteins are encoded by individual subgenomic mRNAs, which are transcribed via a stop-start mechanism resulting in a gradient of messages, with the most abundant messages mapping to the 3′ end of the genome [1], [13]. VSV is potently restricted by RNA silencing [2], [12], [14]. We employed high throughput small RNA sequencing to characterize the vsiRNAs produced during VSV infection of *Drosophila* cells. We found that the majority of VSV vsiRNAs are 21 nt in length (Figure 1B), which is the expected size of Dcr-2 cleavage products that are active in an Ago2-containing RISC. To identify the genomic regions that give rise to these vsiRNAs, we mapped the 21 nt vsiRNAs onto the VSV genome and antigenome sequences, and found that the vsiRNAs are approximately evenly distributed between the genomic and antigenomic strands at a ratio of 1.2∶1. Remarkably, the majority of these vsiRNAs cluster in a discrete ∼1.6 kb region at the 5′ end of the genome (Figure 1C). A similar distribution pattern is observed when the remaining non-21 nt vsiRNAs are mapped onto the VSV genome; however, non-21 nt species are much less abundant (Figure S1A).

To further define the mechanism of virus restriction by antiviral RNAi, we examined the contribution of known components of *Drosophila* RNA silencing pathways to the observed VSV vsiRNA size distributions and genomic mapping patterns. To address vsiRNA biogenesis, we depleted cells of the three somatic RNase III enzymes Dcr-2, Dcr-1 and Drosha, challenged them with VSV, and characterized the vsiRNAs produced. Functional depletion was confirmed by small RNA northern blotting and qPCR (Figure S2). Consistent with our hypothesis that most VSV vsiRNAs are generated by Dcr-2, loss of this factor nearly abolished all vsiRNA biogenesis (14.2-fold decrease) (Figure 1D). The magnitude of this effect is particularly striking when examining the extensively targeted 1 kb region at the 5′ terminus of the genome (Figure 1E, compare black and green traces). In contrast, Dcr-1 and Drosha depletion does not greatly alter VSV vsiRNA levels (Figures 1D–E).

To examine the contribution of RISC loading and stabilization to steady state vsiRNA levels, we analyzed the spectrum of VSV vsiRNAs from cells depleted of the somatic Argonautes Ago2 and Ago1. miRNA RISC component Ago1 has little influence on the vsiRNA size distribution and mapping pattern, as expected (Figures 1F–G). Although depletion of the siRNA pathway slicer, Ago2, does not appear to greatly reduce the bulk levels of 21 nt vsiRNAs (Figure 1F), its effect is clear when the vsiRNAs are plotted against the VSV genome. Loss of Ago2 significantly reduces the steady-state levels of vsiRNAs deriving from the majority of the genome, but has no effect on the abundant vsiRNAs mapping to the extensively targeted region at the 5′ terminus (Figure 1G).

### Dcr-2 Targets VSV-derived DI Hairpins

**Figure 2:**
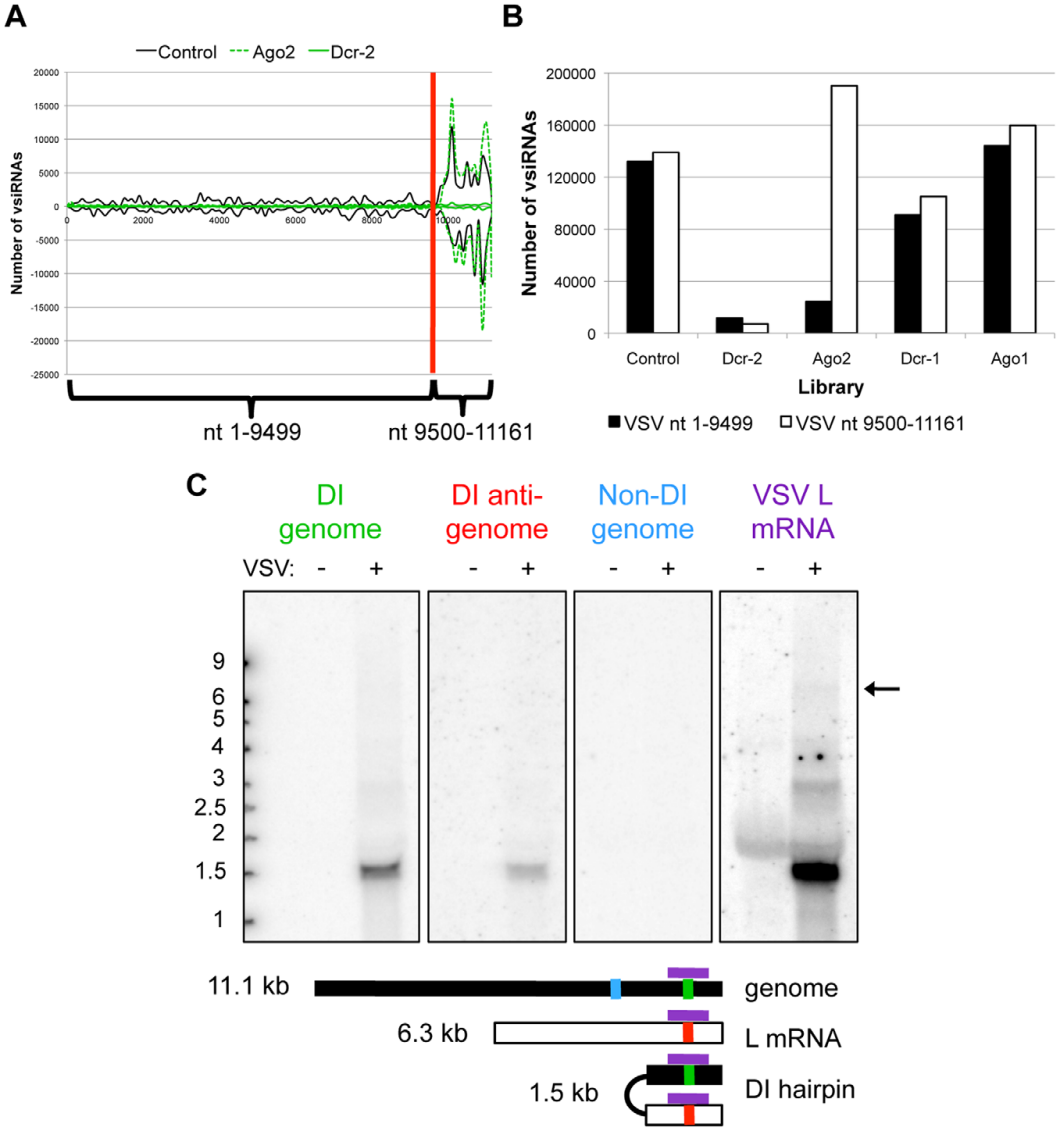
VSV DI particles produce vsiRNAs that are insensitive to RISC depletion. (A) Distribution of vsiRNAs generated from two distinct regions of the VSV genome (nt 1–9499 versus nt 9500–11161) during infection of control cells (black), Ago2-depleted cells (dashed green), and Dcr-2-depleted cells (solid green). (B) Comparison of relative numbers of vsiRNAs mapping to the two regions between libraries, normalized to total library reads. (C) Northern blot analysis of VSV-infected *Drosophila* cells. Strand-specific oligonucleotide probes include (+) sense oligos that recognize genome-sense RNAs either within the heavily targeted region (green) or in the poorly targeted region (blue), and a (−) sense oligo that recognizes antigenomic RNAs within the heavily targeted region (red). A 600 nt probe (purple) recognizes both (+) and (−) sense RNAs within the heavily targeted region. Arrow indicates 6 kb L mRNA (see Figure S3 for darker exposure).

The striking differences in vsiRNA levels derived from the VSV 5′ terminus relative to the rest of the genome led us to dissect this disparity in greater detail. We therefore divided the VSV genome into two regions; nt 1–9499, which is targeted at relatively low levels, and nt 9500–11161, which generates abundant vsiRNAs. VSV vsiRNAs from Dcr-2 and Ago2-depleted cells were then plotted relative to vsiRNAs from control cells (Figure 2A), and the absolute number of vsiRNAs mapping to each region were quantified across VSV libraries (Figure 2B). Although the heavily targeted region only comprises ∼15% of the VSV genome sequence, it produces an equal number of vsiRNAs as the rest of the genome in the control, Dcr-1 and Ago1 depleted libraries (Figure 2A). Loss of Dcr-2 nearly abolishes all vsiRNA biogenesis from both regions (Figures 2A–B). In contrast, knockdown of Ago2 selectively depletes vsiRNAs from nt 1–9499, while the levels of vsiRNAs from nt 9500–11161 are increased by ∼35%, implying that the vsiRNAs derived from the heavily targeted region are not loaded into Ago2 and thus do not require Ago2 for stability (Figures 2A–B). Taken together, these data suggest that although the vast majority of VSV vsiRNAs are produced by Dcr-2, only a particular subset is loaded into Ago2.

These findings also imply that the region that produces abundant vsiRNAs is distinct from the rest of the genome. The heavily targeted region lies within the polymerase (L) coding sequence, but the L mRNA is over 6 kb in length (Figure S3), which cannot account for the discrete targeting of only a ∼1.5 kb stretch. However, VSV is known to generate several types of defective interfering (DI) particles, which contain only small portions of the parental genome and can interfere with parental virus replication [3], [13]. One class of DI particles are termed snapback or hairpin particles and are comprised of perfectly complementary sequences derived from the VSV 5′ terminus [1], [15]. We therefore hypothesized that a snapback DI particle was the source of the abundant VSV vsiRNAs. Strand-specific oligonucleotide probes directed against the heavily targeted 5′ region were used to probe a northern blot of RNA from VSV-infected *Drosophila* cells (Figure 2C, red and green probes). Both the sense and antisense probes recognized a novel ∼1.5 kb band, while an additional probe that recognizes a sequence upstream of the heavily targeted region did not yield a signal (Figure 2C, blue probe). An internally labeled PCR probe designed to detect the L mRNA also recognizes the putative ∼1.5 kb DI RNA, which is present at much higher levels than the L transcript (arrow, Figures 2C, S3). These data suggest that the extensive targeting of the VSV 5′ terminal region may be due to the presence of a highly expressed replicating RNA that is likely a self-complementary DI particle.

### RVFV Infection Generates Abundant vsiRNAs from Genomic and Antigenomic RNA Strands

**Figure 3:**
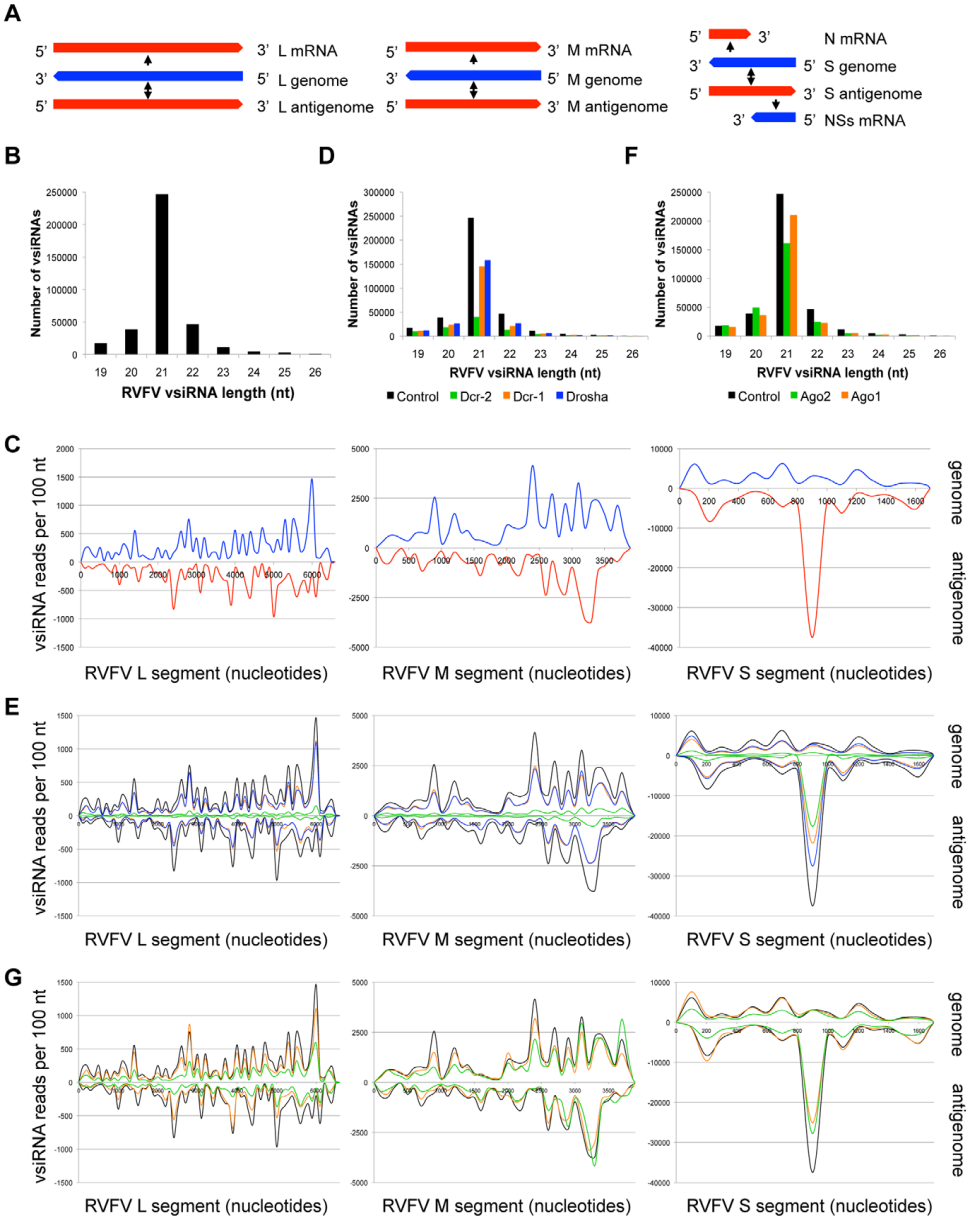
Both the genomic and antigenomic RNA strands of the arbovirus RVFV generate vsiRNAs. (A) RNA species produced during RVFV infection. (−) strand genomic segments and mRNAs are depicted in blue, (+) strand antigenomes and mRNAs in red. (B) RVFV vsiRNA size distribution (control library). (C) Distribution of 21 nt RVFV vsiRNAs across the three viral genomic segments. vsiRNAs mapping to genomic strand are depicted in blue, antigenomic strand in red. (D) RVFV vsiRNA size distribution between libraries depleted of RNase III enzymes. (E) Effect of RNase III enzyme depletion on 21 nt RVFV vsiRNAs. vsiRNAs from control (black), Dcr-1 (orange), Dcr-2 (green) and Drosha (blue) depleted cells are compared. (F) RVFV vsiRNA size distribution between libraries depleted of Argonaute proteins. (G) Effect of Argonaute depletion on 21 nt RVFV vsiRNAs. vsiRNAs from control (black), Ago1 (orange), and Ago2 (green) depleted cells are compared. See also Figures S1, S2.

RVFV is a member of the *Bunyaviridae* family, one of the main classes of medically relevant arboviruses. RVFV carries a ∼12 kb trisegmented negative sense genome comprised of the large (L) medium (M) and small (S) segments (Figure 3A). While both the L and M segments encode transcripts in the sense orientation, the S segment encodes two distinct gene products using an ambisense coding strategy [4], [16]. RVFV infects Drosophila and is restricted by the RNA silencing machinery ([1], [17], data not shown). We found that the majority of RVFV vsiRNAs are 21 nt in length (Figure 3B). The vsiRNAs derived from the L and M segments are present in approximately equal ratios (1.3∶1) between the (+) and (−) strands, and are distributed in a uniform manner across the genomic segments (Figures 3C, S1D). This suggests that the L and M segments are targeted at the level of the replicating viral dsRNA. In contrast, the S segment contains a vsiRNA hotspot within a particular region of the antigenome, suggesting a distinct mode of recognition or processing (Figure 3C).

To determine whether RVFV is recognized and processed by the siRNA pathway, RVFV vsiRNA size classes and genomic mapping were characterized following depletion of RNA silencing components. Functional depletion was confirmed by small RNA northern blotting (Figure S2). Loss of Dcr-2 affects all vsiRNA size classes, most notably the levels of 21 nt RVFV vsiRNAs (Figure 3D). The Dcr-2 dependence of RVFV vsiRNAs is particularly evident when the vsiRNAs are mapped onto the viral genomic segments (Figure 3E; compare black and green traces). Knockdown of miRNA components Dcr-1 and Drosha slightly suppresses vsiRNA biogenesis, albeit to a lesser degree than Dcr-2 depletion. Similar to Dcr-2, loss of Ago2 leads to a reduction in 21 nt vsiRNAs (Figures 3F–G). Ago1 depletion slightly reduces vsiRNA levels across all size classes (Figures 3F–G), but its effect on the 21 nt species is not as strong as loss of Ago2.

### RVFV Contains a Putative Intergenic RNA Hairpin

**Figure 4:**
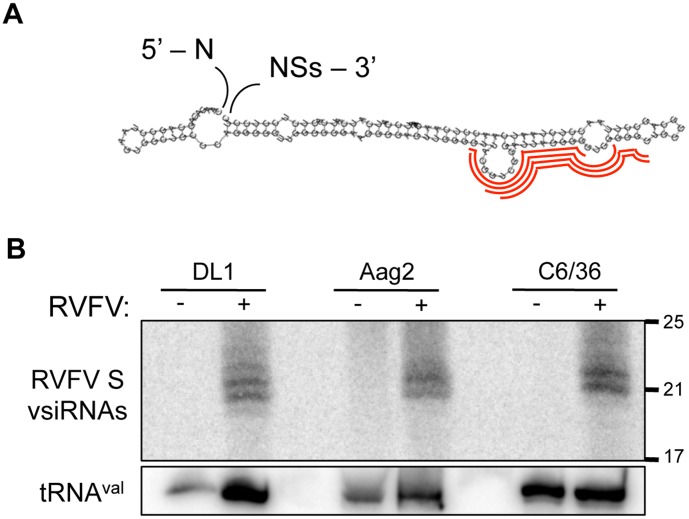
A putative hairpin within the RVFV S segment generates abundant vsiRNAs in *Drosophila* and mosquito cells. (A) RNA secondary structure prediction of S segment IGR. The highly abundant vsiRNAs are mapped in red. (B) Northern blot analysis of RVFV-infected *Drosophila* DL1 cells, *Aedes aegypti* Aag2 cells, and *Aedes albopictus* C6/36 cells, probed for the S segment stem loop vsiRNAs and tRNA^val^ as a loading control.

While we noted that the RVFV L and M segment vsiRNAs were evenly derived from the genome and antigenome, and evenly distributed across their lengths, the S segment appeared to be uniquely targeted. A large fraction (37.8%) of total RVFV vsiRNAs mapped to a particular region in the S segment antigenome (Figure 3C). Specifically, two 21 nt vsiRNAs and two 22 nt vsiRNAs derived from within the S segment intergenic region (IGR) are extremely abundant in the library. Interestingly, the S segment IGR is thought to fold into an RNA hairpin in several bunyaviruses [1], [16], and RNAfold predicts the presence of a hairpin within this sequence (Figure 4A). The abundant S segment vsiRNAs map within the predicted stem region (Figure 4A, red) and are readily detected by small RNA Northern blotting of infected *Drosophila* cells (Figure 4B). To determine whether this putative hairpin is targeted during infection of mosquitoes, which are responsible for natural transmission of the virus, we challenged two mosquito cell lines with RVFV: *Aedes aegypti* Aag2 cells and *Aedes albopictus* C6/36 cells. Small RNA northern blotting revealed that the vsiRNAs derived from the putative intergenic hairpin are highly abundant in RVFV-infected Aag2 and C6/36 cells but not uninfected cells (Figure 4B). Although C6/36 cells have been shown to have a defective siRNA pathway [2], [18], the biogenesis of these abundant vsiRNAs is unaltered in these cells, which may suggest that other cellular or viral proteins are involved in their production. This is also supported by our observation that knockdown of Dcr-2 does not completely abolish their production (Figure 3E). Thus, in the case of RVFV, the most abundant vsiRNAs are not produced from a dsRNA replication intermediate, but instead derive from a discrete intergenic region that likely forms a structured viral RNA hairpin. In other bunyaviruses, S segment intergenic hairpins play essential roles in viral RNA replication, indicating the necessity to conserve this structure despite the presence of an antiviral RNAi response [1], [16].

### The DCV vsiRNA Distribution Reflects Extensive Targeting of Single Stranded Genomic RNA

**Figure 5:**
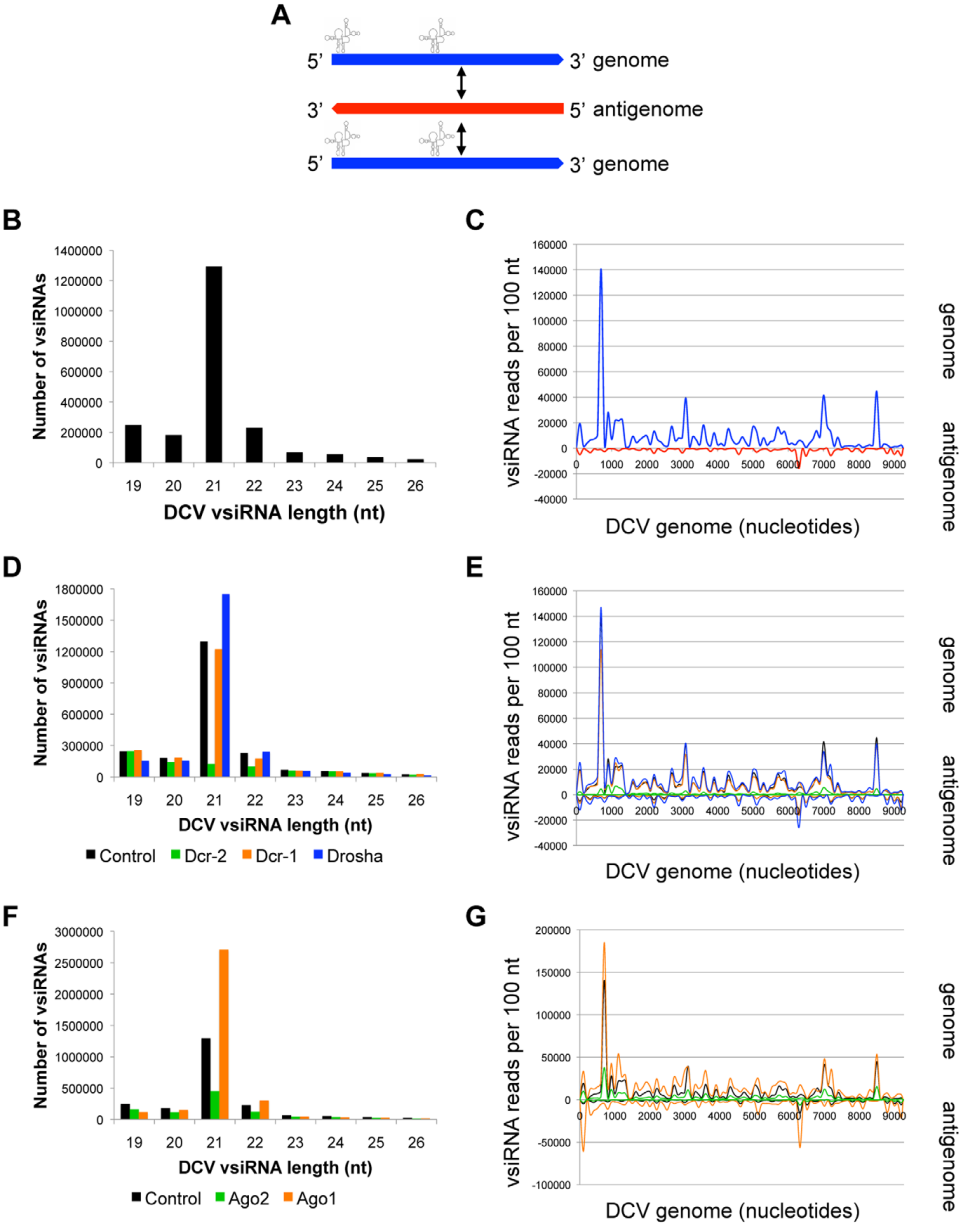
DCV genomic strand RNA is preferentially targeted by antiviral RNAi. (A) RNA species produced during DCV infection. (+) strand genome is depicted in blue, (−) strand antigenome in red. (B) DCV vsiRNA size distribution (control library). (C) Distribution of 21 nt DCV-derived vsiRNAs across the viral genome. vsiRNAs mapping to genomic strand are depicted in blue, antigenomic strand in red. (D) DCV vsiRNA size distribution between libraries depleted of RNase III enzymes. (E) Effect of RNase III enzyme depletion on 21 nt DCV vsiRNAs. vsiRNAs from control (black), Dcr-1 (orange), Dcr-2 (green) and Drosha (blue) depleted cells are compared. (F) DCV vsiRNA size distribution between libraries depleted of Argonaute proteins. (G) Effect of Argonaute depletion on 21 nt DCV vsiRNAs. vsiRNAs from control (black), Ago1 (orange), and Ago2 (green) depleted cells are compared. See also Figures S1, S2.

As a natural *Drosophila* pathogen that has co-evolved with the host, we hypothesized that the antiviral pathway may be uniquely poised to restrict DCV replication. Indeed, RNAi pathway components are potently antiviral against DCV [2], [19], [20]. DCV encodes a 9.3 kb positive-stranded genome that contains two internal ribosome entry sites (IRESs) that are highly structured, and no subgenomic transcripts (Figure 5A) [4], [5], [21]. Analysis of the small RNAs from DCV-infected *Drosophila* cells revealed that vsiRNAs are predominantly 21 nt in length (Figure 5B). Strikingly, the majority of 21 nt (Figure 5C) and non-21 nt (Figure S1B) DCV vsiRNAs map to the genomic (+) strand (87.4%). The preferential targeting of genomic RNA is consistent both with previous studies of DCV vsiRNA patterns in a different *Drosophila* cell line [6]–[11], [22], and with infection of adult flies by wild-type Flock House virus (FHV), another insect pathogen [1], [12], [23]. The unequal skewing of DCV vsiRNAs to the (+) strand could be a result of Dcr-2 processing of structured regions and hairpins within the abundant single-stranded genomic (+) strand RNA, or the skewing could be due to enhanced stability of (+) strand-derived vsiRNAs processed from dsRNA replication intermediates. Although some of the (+) strand DCV vsiRNAs may simply be degradation products of the abundant genome strand RNAs, the majority are 21 nt in length, suggesting that they are bona-fide Dcr-2 products. Due to their extensive secondary structure, we expected the two viral IRES structures to be extensively targeted by the antiviral machinery. However, this is not the case; although several regions of the DCV genome appear to be “hot spots” for vsiRNA biogenesis, these regions do not fall within the IRES sequences.

To further study the production of DCV vsiRNAs, we depleted the enzymes involved in RNA silencing and confirmed functional depletion by small RNA northern blotting (Figure S2). Knockdown of Dcr-2 leads to a striking reduction of 21 nt vsiRNAs, demonstrating that the skewed (+) strand bias is not simply due to random degradation of genomic RNAs (Figures 5D, E). In contrast to Dcr-2, depletion of miRNA pathway components Dcr-1 and Drosha do not affect the spectrum of 21 nt DCV vsiRNAs (Figures 5D–E).

Next, we assessed the role of RISC components in generating the DCV vsiRNA spectrum. Knockdown of Ago2 results in a reduction in all vsiRNA size classes, particularly 21 nt vsiRNAs, while loss of Ago1 has variable effects on each size class (Figure 5F). Although neither knockdown affects the vsiRNA distribution pattern across the DCV genome, the magnitudes of vsiRNA hotspots are reduced by Ago2 depletion (Figure 5G), suggesting that 21 nt DCV vsiRNAs are loaded into Ago2, and the loss of this factor leads to their destabilization. Altogether, these data suggest that DCV vsiRNAs are predominantly generated and stabilized by the canonical siRNA pathway components Dcr-2 and Ago2, consistent with the pathway’s known role in antiviral defense. However, rather than the viral dsRNA intermediate, it is the viral genomic RNA that is responsible for generating the majority of DCV vsiRNAs.

### DCV Infection Alters Dcr-2 Processing

**Figure 6:**
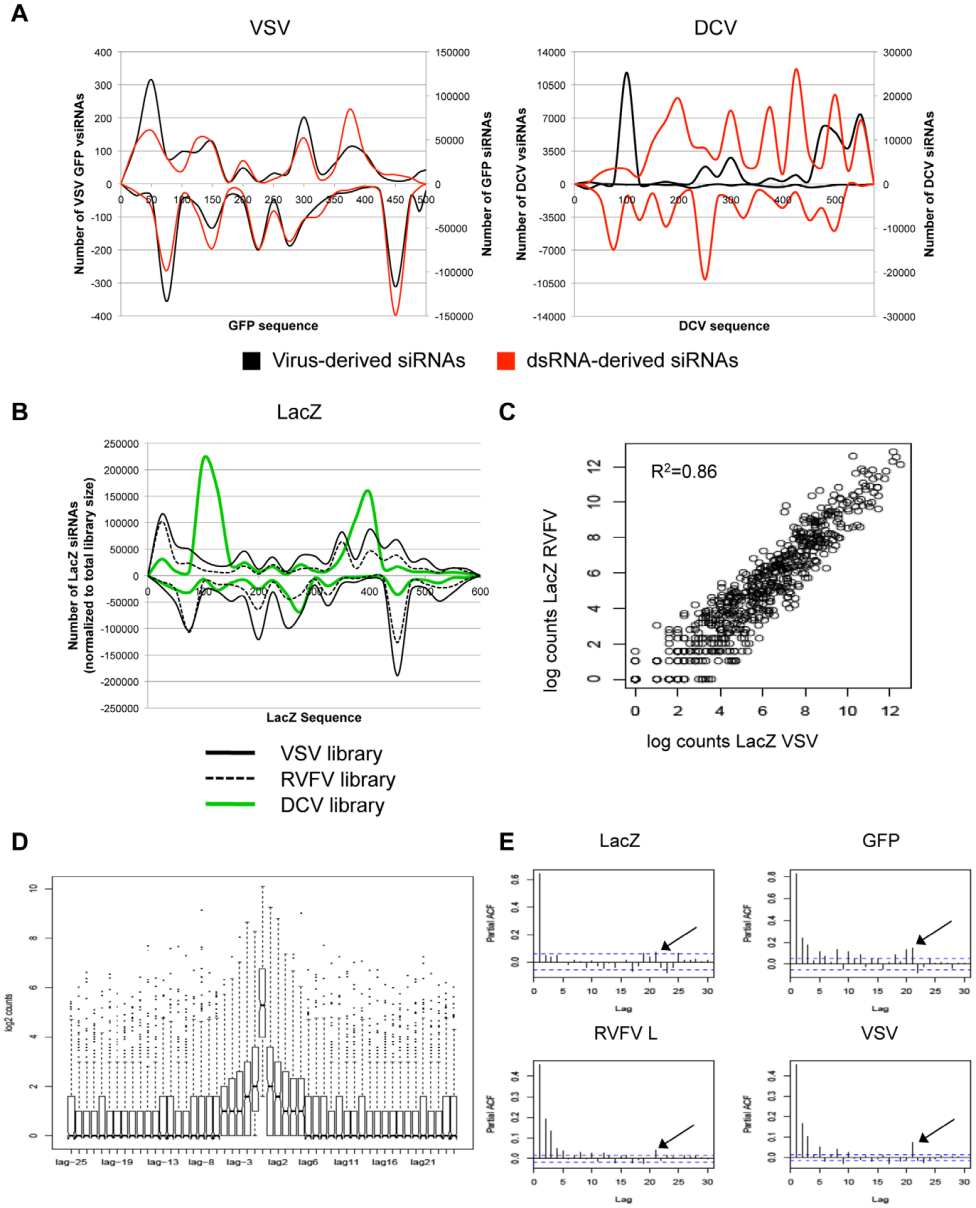
Viral and synthetic dsRNA processing yields reproducible patterns of small RNA abundance. (A) Distribution of vsiRNAs compared to synthetic dsRNA-derived siRNAs corresponding to VSV (left) or DCV (right). Virus-derived siRNAs are shown in black, synthetic dsRNA-derived siRNAs are shown in red. (B) Distribution of siRNAs generated from LacZ dsRNA in cells infected with VSV (solid black), RVFV (dashed black) or DCV (green). (C) Reproducibility of Log_2_-transformed 21 nt read counts from LacZ dsRNA present in VSV- or RVFV-infected cells. (D) Lagged response data for RVFV M/L segment peaks. Distribution of logged counts upstream (positive lags) and downstream (negative lags) of position 1 of the peak siRNA sequence are shown. (E) Partial autocorrelation functions of four sequences; exogenous LacZ, exogenous GFP, RVFV L segment, VSV nt 1–9000. Dotted lines indicate the 95% significance level. Black arrows indicate additional regions of significant autocorrelation at 21 nt.

Our observation that VSV and RVFV vsiRNAs are derived from genomic and antigenomic RNA strands in approximately equal ratios led us to postulate that this distribution pattern reflects targeting of dsRNA replication intermediates. In contrast, the DCV vsiRNA distribution is strikingly different than that of VSV or RVFV; the majority of DCV vsiRNAs map exclusively to the genomic RNA strand, suggesting that the dsRNA replication intermediate is not the primary Dcr-2 target. Interestingly, DCV encodes an RNAi suppressor, DCV-1A, which binds dsRNA and is therefore thought to protect the DCV dsRNA replication intermediate during infection [4], [6], [20]. Therefore, we reasoned that the skewed vsiRNA distribution pattern may be due to the presence of DCV-1A; if the viral dsRNA replication intermediate is protected by binding to DCV-1A, the antiviral machinery may be forced to target other viral RNA species. Neither VSV nor RVFV are known to carry a suppressor of RNAi, which could, in part, explain the ability of the RNAi machinery to target their dsRNA replication intermediates. We thus reasoned that in the absence of infection, synthetic dsRNAs bearing viral sequences would be processed by the RNAi pathway to yield siRNAs that are distributed equally between the positive and negative strands, We examined the siRNAs produced in *Drosophila* cells bathed with an *in vitro* synthesized GFP dsRNA, and compared them to the vsiRNAs generated from VSV-encoded GFP, which is expressed off of a subgenomic transcript during infection. Indeed, the distribution pattern generated by bathing cells in GFP dsRNA overlapped the distribution of VSV-derived GFP vsiRNAs and was approximately equally distributed between both RNA strands (Figure 6A, left). We then compared the relative abundance of siRNAs generated from cells treated with a DCV-specific dsRNA to the vsiRNA distribution pattern from the corresponding DCV genomic region. The synthetic dsRNA bearing DCV sequences generated siRNAs that mapped approximately equally to both strands, in contrast to the DCV vsiRNAs produced during infection, which are heavily skewed to the genomic (+) strand (Figure 6A, right). These results support the hypothesis that VSV (and by extension, RVFV) dsRNA replication intermediates are targeted during vsiRNA biogenesis, while DCV replication intermediates are not the primary targets of the antiviral machinery.

To examine the potential role that DCV-1A may play in influencing patterns of small RNA abundance, we asked whether siRNA distributions from synthetic dsRNAs are skewed during DCV infection, since DCV-1A could bind and protect them from Dcr-2 processing. To test this, we examined the siRNAs produced from LacZ dsRNA, which was used as a non-targeting control for the VSV, RVFV and DCV libraries, and monitored the distribution pattern of siRNAs generated from this exogenous dsRNA. While the patterns of LacZ siRNAs produced during VSV and RVFV infections are very similar (Figure 6B, solid and dashed black lines), LacZ siRNAs produced during DCV infection display a distinct distribution (Figure 6B, green). These results support the notion that the RNAi suppressor protein DCV-1A influences Dcr-2 targeting during DCV infection, and likely explain the unequal distribution pattern of DCV vsiRNAs during infection.

### Viral and Synthetic dsRNAs Generate Reproducible Patterns of Small RNAs

We noted that the individual peaks and troughs within the vsiRNA mapping patterns were consistent between experiments, and the distribution pattern of LacZ siRNAs produced in VSV and RVFV infected cells appeared to be highly similar (Figure 6B), suggesting that the distributions are a reproducible signature of cellular targeting of RNA precursors rather than a result of stochastic events. To quantify these observations, we compared the abundance of LacZ dsRNA-derived siRNAs sequenced from independent libraries. Raw reads for each individual LacZ siRNA from the control VSV library were plotted against the LacZ siRNA reads from the control RVFV library (data not shown). The untransformed data exhibits increased variance at higher read values, indicative of heteroscedasticity that can be stabilized by proper transformation of the data. A Log_2_ transformation was used for statistical inference (Figure 6C). Log_2_ transformed LacZ siRNA counts show a significant degree of reproducibility between libraries (R^2^ = 0.86; Figure 6C). These comparative analyses reveal that a given dsRNA sequence generates a reproducible spectrum of siRNAs when targeted by the *Drosophila* RNA silencing machinery.

To dissect the specificity determinants of precursor RNAs targeted by Dcr-2 that reproducibly give rise to high or low abundance small RNAs, we began by examining the local distribution of abundant small RNA peaks. We borrowed a technique from time series analysis in which responses at time t are compared to the immediately preceding responses; time t-1 is designated as lag1, time t-2 as lag2, etc [13], [24]. Thus, read counts for 21-mers starting at each position in the precursor molecule were compared with counts at neighboring sites. We identified peaks of small RNA read counts in the data and examined the surrounding positions for correlation. From a 6.7 kb subset of the RVFV dataset (L and M segments), the distribution of counts around the peaks shows clear clustering of 21-mers, with significantly higher than background counts extending five nucleotides up- and downstream of the peak (Figure 6D). To determine whether this phenomenon could be extended to other small RNA precursors, we computed the partial autocorrelation functions of the abundant small RNAs from four sequences, including two viral genomes and two exogenous dsRNAs (Figure 6E). Autocorrelations can be used to assess whether the abundance of one small RNA is related to the abundance of an adjacent small RNA. For each precursor sequence, high autocorrelation is seen at the lag 1, 2, and 3 positions (Figure 6E). In addition, we observed a 21 nt lag that shows significant autocorrelation (Figure 6E, black arrows), which suggests that at least some of the small RNAs are processively cleaved from precursor molecules at 21 nt intervals, a signature of Dcr-2 processing.

### RNA Transcripts Produced by the DNA Virus VACV are Dcr-2 Targets

**Figure 7:**
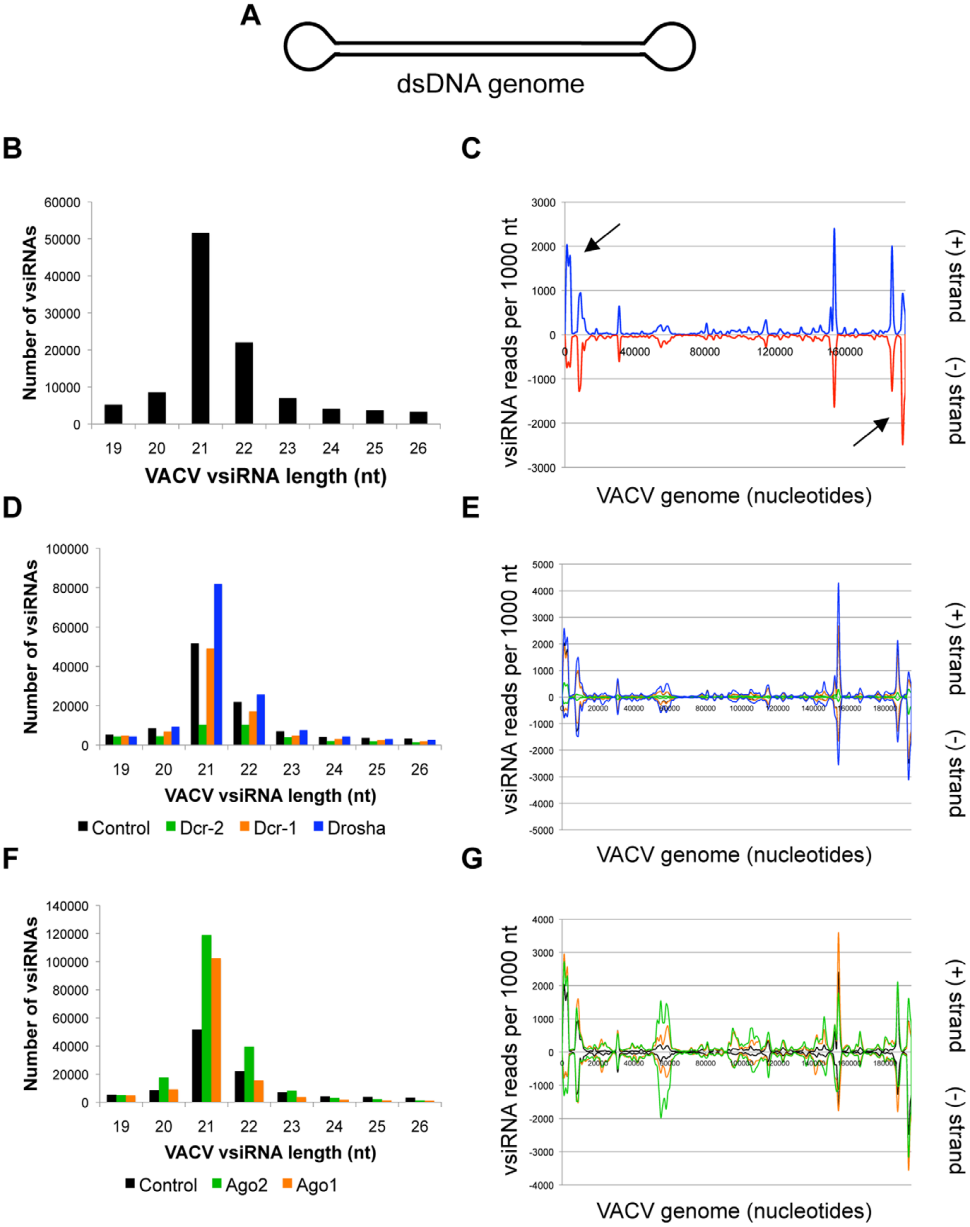
RNA transcripts produced by VACV are targeted by the *Drosophila* RNA silencing pathway. (A) The VACV genome is a dsDNA molecule with covalently closed identical termini. (B) VACV vsiRNA size distribution (control library). (C) Distribution of 21 nt VACV vsiRNAs across the viral genome. vsiRNAs mapping to the (+) strand are depicted in blue, (−) strand in red. Black arrows mark genomic termini. (D) VACV vsiRNA size distribution between libraries depleted of RNase III enzymes. (E) Effect of RNase III enzyme depletion on 21 nt VACV vsiRNAs. vsiRNAs from control (black), Dcr-1 (orange), Dcr-2 (green) and Drosha (blue) depleted cells are compared. (F) VACV vsiRNA size distribution between libraries depleted of Argonaute proteins. (G) Effect of Argonaute depletion on 21 nt VACV vsiRNAs. vsiRNAs from control (black), Ago1 (orange), and Ago2 (green) depleted cells are compared. See also Figures S1, S2.

VACV is a poxvirus closely related to Variola virus, the causative agent of smallpox. VACV was selected as the sole DNA virus in the panel because it replicates exclusively in the cytoplasm (in contrast to other families of DNA viruses) and thus might be unusually sensitive to cytoplasmic small RNA silencing pathways [25]. The VACV genome is a ∼200 kb dsDNA molecule with covalently closed terminal loops (Figure 7A). VACV cannot undergo a complete replication cycle in *Drosophila* cells but can infect the cells and express early gene products [26]. We found that VACV vsiRNAs are produced in infected *Drosophila* cells and are predominantly 21 nt in length (Figure 7B). The 21 nt vsiRNAs map to both the Watson and Crick strands of the virus, and are particularly concentrated at the extreme genomic termini (Figure 7C, black arrows). The non-21 nt vsiRNAs display a similar distribution pattern, and also map abundantly to the genomic termini (Figure S1C). We also noted several abundant peaks of vsiRNAs that were distinct from the genomic termini. One such region likely generates vsiRNAs through the targeting of dsRNA produced by bidirectional transcription; a more detailed diagram of the hot spot is provided in Figure S4A.

We reasoned that VACV vsiRNAs may be generated through two mechanisms: bidirectional transcription at a particular locus could yield dsRNA that is processed into vsiRNAs, or structured regions within single transcripts could be targeted by the RNA silencing machinery or other cellular nucleases. Again, we depleted the enzymes involved in RNA silencing and confirmed functional depletion by small northern blotting (Figure S2). We found that depletion of Dcr-2 led to a significant reduction in the 21 nt species, with a more modest effect on the other size classes (Figure 7D). When mapped against the viral genome, the role of Dcr-2 is clear; the peaks of abundant 21 nt vsiRNAs are nearly abolished in the absence of Dcr-2 (Figure 7E, compare black and green traces). Knockdown of miRNA component Dcr-1 does not strongly deplete any vsiRNA size classes or influence their genomic mapping pattern, but depletion of Drosha leads to an increase in vsiRNA accumulation, particularly of the 21 nt species (Figure 7E). We next assessed the contribution of the Argonautes Ago1 and Ago2 to VACV vsiRNA levels. Unexpectedly, loss of siRNA RISC component Ago2 increases both 21 nt and non-21 nt VACV vsiRNAs (Figures 7F–G). Ago1 depletion also increases the levels of 21 nt vsiRNAs, while the non-21 nt classes appear to be fairly unaffected (Figures 7F–G).

### Terminal Repeats in the VACV Genome Produce Abundant vsiRNAs

**Figure 8:**
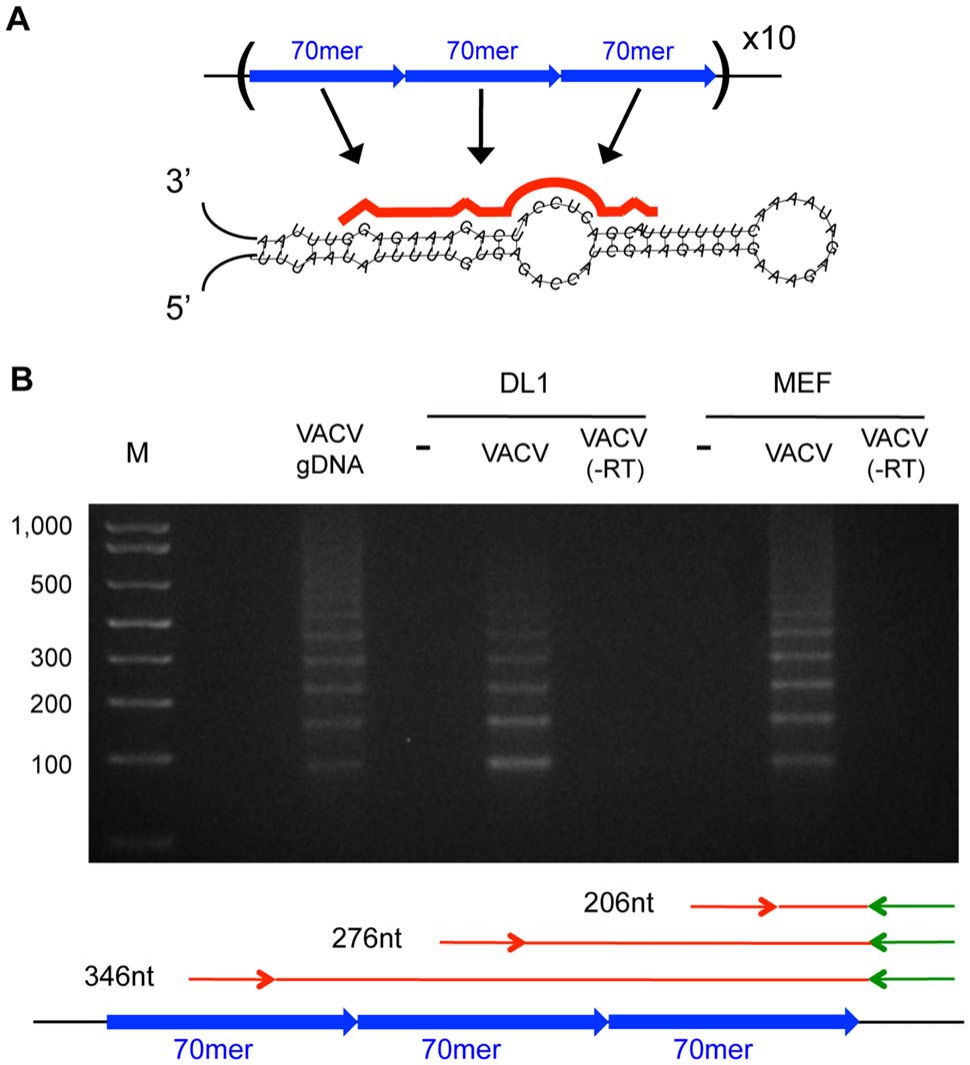
VACV terminal repeat-derived vsiRNAs are derived from long, repeat-containing precursors. (A) RNA secondary structure prediction of one of sixty 70-mer repeats located at the genomic termini. The abundant repeat-associated VACV vsiRNA is mapped in red. See also Figure S4. (B) Expression analysis of VACV terminal repeat-associated transcripts in *Drosophila* DL1 cells and mouse embryonic fibroblasts (MEFs) by RT-PCR. The forward primer (red) lies within the 70-mer repeat sequence, while the reverse primer (green) binds a unique sequence outside of the repetitive region. The banding pattern of PCR products reflects the amplification of variable numbers of 70-mer repeats, as depicted in the diagram. M = DNA ladder.

As noted above, a significant fraction of the VACV vsiRNAs map to the two genomic termini (Figure 7C, black arrows). Each VACV genomic terminus is identical and contains tandem repeats of a 70 nt element, with a total of 30 copies per terminus [25]. This region is devoid of open reading frames and instead is thought to play a role in DNA replication [25]. Examination of the vsiRNAs derived from these elements revealed that the majority are 21 or 22 nt in length and align to a discrete region within the 70 nt repeat sequence (Figure S4B). Upon knockdown of Dcr-2, but not Dcr-1, the most abundant 21 nt vsiRNA was depleted (Figure S4C). In contrast, the most abundant 22 nt species appear to require both Dcr-2 and Dcr-1 for biogenesis, since the 22 nt vsiRNA levels are moderately depleted in both Dcr-2 and Dcr-1 deficient cells (Figure S4C). Loss of Ago2 led to a reduction in both the 21 and 22 nt repeat-derived vsiRNAs, while Ago1 depletion only affected the levels of the 22 nt species (Figure S4D). We modeled the predicted secondary structure of one 70 nt repeat using RNAfold, and mapped the most abundant 21 nt VACV vsiRNA onto the structure (Figure 8A; red). The predicted structure is a stem-loop reminiscent of a pre-miRNA; the VACV vsiRNAs map to the stem region.

These *in silico* results suggested that transcription of the VACV tandem repeats gives rise to long, structured RNAs that serve as siRNA and miRNA pathway substrates. Indeed, we found that this region is expressed in *Drosophila* and mammalian cells (Figure 8B). Moreover, the 70-mers do not appear to be expressed individually but instead are present in longer transcripts containing multiple copies of the repeat, since products corresponding to at least 5 copies of the repeat can be amplified by RT-PCR (Figure 8B). The role of this highly structured RNA that contains no known ORFs, and how dicing of this transcript impacts poxvirus replication, is unknown.

## Discussion

The results of this study serve to reinforce the notion that a diverse set of viral RNA structures are produced during infection, and that cells are capable of differentially recognizing and targeting these structures. Although the viruses in our panel display striking differences in the spectrum of vsiRNAs produced during infection, in each case the majority of the vsiRNAs are Dcr-2 cleavage products. Therefore, Dcr-2 is the major nuclease, recognizing and processing a wide range of substrates, an idea that has recently been solidified by the discovery that endogenous, Dcr-2-dependent siRNAs derive from a variety of precursors, including structured RNAs and dsRNA formed during bidirectional transcription [1]. However, in some cases, we found that the levels of vsiRNAs were partially dependent on components of the miRNA machinery as well as the siRNA pathway. For instance, knockdown of Drosha or Ago1 leads to a slight increase in vsiRNAs for several viruses (Figures 1, 5 and 7). An increase in FHV vsiRNAs upon Ago1 depletion has also been observed by Flynt and colleagues [27]. One hypothesis to explain these effects is that the miRNA pathway normally represses expression of a pro-viral factor. Impaired miRNA pathway function would therefore lead to increased expression of the pro-viral factor, leading to enhanced viral replication, which provides more viral RNA substrates to the Dcr-2-driven RNAi pathway. Alternatively, cross-loading of vsiRNAs into an active Ago1 complex, which is a common fate for several endogenous siRNAs [28], might also explain the increase in viral replication and vsiRNAs production in the absence of Ago1. Cross-loading into Ago1 may also explain the differential stability of VSV DI hairpin-derived vsiRNAs (Figure 2). However, detailed future studies are necessary to dissect the role of the miRNA pathway in vsiRNA biogenesis.

Our data demonstrate that the viral RNAs targeted by the cellular RNAi machinery fall into several general categories including dsRNA intermediates (RVFV L and M segments, VSV), genomic RNAs (DCV), and unique, heavily targeted predicted hairpins (RVFV S segment; VACV tandem repeats). Moreover, the distribution pattern of vsiRNAs produced from viral dsRNA can mirror the distribution observed from a synthetic dsRNA bearing the same sequence (Figure 6). Thus, the vsiRNA levels that we have characterized are governed, at least in part, by properties of the RNAs themselves.

These findings complement and expand upon previous studies examining arbovirus-derived vsiRNAs. Deep sequencing of vsiRNAs from various mosquito species and mosquito-derived cell lines has revealed that vsiRNAs produced from Sindbis virus, Semliki Forest virus, and Dengue virus type 2 are present in approximately equal ratios (∼1.5∶1 genome:antigenome) and are distributed along the length of the genome [10], [18], [29]. These patterns resemble our results for RVFV and VSV, suggesting that the targeting of dsRNA replication intermediates is a common mechanism of antiviral RNAi that is effective against multiple arboviruses. In fact, a study by Mueller and colleagues previously described vsiRNA profiles of VSV infection of adult flies; the vsiRNAs map approximately equally to both strands, suggesting that the viral replication intermediate is targeted [14]. These results demonstrate that the antiviral RNAi pathway operates similarly in *Drosophila* cells and adult flies, supporting the notion that the observations made in cell culture reflect the function of the RNAi pathway in vivo.

To our knowledge, this study is the first to investigate the production of vsiRNAs from a DNA virus. Many mammalian DNA viruses, most notably Herpesvirus family members, co-opt host RNA silencing machinery in order to express virus-encoded miRNAs, which are transcribed as stem loop precursors and require processing by the host miRNA biogenesis pathway [30]. VACV is not thought to encode viral miRNAs, since its exclusively cytoplasmic replication precludes access to the nuclear miRNA processing machinery. However, we identified virus-derived small RNAs produced during VACV infection. The most prominent group were derived from structured hairpins encoded by terminal repeat sequences (Figure 8) and provide the first evidence for putative VACV-encoded small RNAs that may play a regulatory role during infection. We also identified additional hotspots which have yet to be fully characterized. Given the large number of insect-borne poxviruses [31], it is likely that poxviruses have evolved strategies to harness or counteract insect RNA silencing machinery. Indeed, we recently found that the VACV-encoded poly(A)polymerase VP55 can target endogenous miRNAs for degradation both in insects and mammals [32].

Another important facet of the antiviral RNAi response is the ability of the virus to overcome the restrictive pressure placed on it by the pathway. Thus, many pathogenic plant- and insect-specific viruses encode suppressors of silencing [4]. Among the viruses queried in this study, DCV is the only pathogenic insect virus, and is known to carry DCV-1A, a protein suppressor of RNAi that binds long dsRNA and reduces the accessibility of dsRNAs to Dcr-2 [20]. The insect virus FHV also carries a suppressor, B2, which binds siRNAs and long dsRNAs [4]. FHVΔB2, a strain that does not express B2, produces approximately equal (+) and (−) sense vsiRNAs in wild-type flies and cells [23], [27], [33]. However, the vsiRNAs produced during wild-type FHV infection of wild-type flies are heavily skewed toward the genomic strand [22], [23], similar to our results with DCV (Figures 5–6). Interestingly, we and others have found that West Nile virus (WNV), a human arbovirus, also displays a vsiRNA distribution that is skewed toward the genomic strand [7] (Sabin and Cherry, unpublished observations). Consistently, a putative RNA-based suppressor of dicing, encoded within the WNV 3′ UTR, was recently identified [34]. This supports the hypothesis that in the presence of an RNAi suppressor that impairs the pathway at the level of dicing, vsiRNAs are skewed towards the genomic RNA strand, which is more abundant than the minus strand.

Although many insect viruses encode protein suppressors of RNA silencing, such as DCV-1A, our identification of unique, heavily targeted RNAs such as the VSV DI particles (Figure 2) and the putative RVFV S segment intergenic hairpin (Figure 4) raises the possibility that some viruses may employ RNA decoys as a mechanism to evade the antiviral RNAi machinery. It will therefore be of interest to compare the role of the RVFV hairpin in mammalian versus insect infections, since the mammalian RNAi pathway is not intrinsically antiviral. In the case of VSV, the DI snapback particles may serve as an RNA decoy to direct Dcr-2 activity away from genome targeting. Since the DI hairpins are targeted by Dcr-2, but the resulting vsiRNAs are not stabilized by Ago2 (Figure 2B), they likely cannot target RISC to viral transcripts. Thus, a redirection of Dcr-2 to process substrates that will not go on to degrade additional viral RNAs may allow the virus to partially evade the antiviral activity of the siRNA pathway. These findings are supported by the study by Mueller et al., which found that when high levels of VSV replication were achieved in *AGO2-*deficient flies, creating a favorable condition for DI particle formation, a preference for 5′ end targeting was observed [14]. However, in wild type cells or flies with lower levels of infection, that disfavor the production of DI particles, the vsiRNA distribution was even across the genome [14]. This suggests that the over-replication of DI particles represents an active immune evasion strategy for VSV. By diverting recognition and cleavage to decoy transcripts, a virus may reduce the efficiency of pathway targeting of other essential viral RNAs. Therefore, RNAi pathway impairment using RNA decoys could operate as a novel nucleic acid-based immune evasion mechanism in insects, serving as an additional layer of host antagonism by viral pathogens.

## Materials and Methods

### Cells and Viruses

*Drosophila* DL1 cells were grown and maintained as previously described [35]. *Aedes aegypti* Aag2 cells [36] were grown and maintained at 25°C in complete Schneider’s Drosophila media (Gibco). *Aedes albopictus* C6/36 cells were obtained from ATCC, grown and maintained at 25°C in L-15 medium (Gibco). Mouse embryonic fibroblasts (MEFs) [26] were grown in DMEM (Gibco). RVFV strain MP12 was grown in Vero cells [37]. VSV-eGFP (gift from J. Rose) was grown in BHK cells as described [38]. DCV was grown and purified as described [35]. Vaccinia strain vPRA13 was grown in HeLa S3 suspension cells [39].

### RNAi and Viral Infections

RNAi was performed as previously described [12]. Cells were infected for 4 days as follows: DCV MOI = 7; RVFV MOI = 17; VACV MOI = 383; VSV MOI = 4. Aag2 and C6/36 cells were infected with RVFV MOI = 12.5 for 4 days. MEFs were infected with VACV MOI = 1 for 8 hours.

### RNA Analysis

Total RNA was extracted and analyzed by traditional northern blotting, small RNA northern blotting and RT-PCR as previously described [12]. Knockdown of RNAi machinery was quantified using a SYBRGreen (Applied Biosystems)-based qPCR assay using oligodT-primed cDNA. Oligonucleotide probes and primers are listed in Supporting Information.

### Small RNA Library Generation

40 µg of total RNA was separated on a 15% TBE-urea gel and small RNAs ∼15–29 nt were excised and eluted. Following ethanol precipitation, small RNA-seq libraries were produced using the Small RNA Sample Prep v1.5 kit (Illumina, San Diego, CA) as per manufacturer’s instructions.

### Accession Numbers

All raw small RNA sequence data was deposited in Gene Expression Omnibus (GEO) under the accession GSE43031. This includes 24 total sequence datasets that include control as well as Dcr-1, Dcr-2, Drosha, Ago1, and Ago2 knockdowns for all four viruses.

### Bioinformatics and Computational Analysis

Detailed descriptions of small RNA mapping procedures and vsiRNA profile generation are found in Supporting Information. The genomic location of individual vsiRNAs or siRNAs relative to viral genomes or synthetic dsRNAs are found in Tables S1, S2, S3, S4, and S5. Time series analyses were performed with the R environment [40], and autocorrelation analysis was performed with the MASS package [24].

## Supporting Information

Figure S1 **Related to** **Figures 1** **,** **3** **,** **5** **, and** **7** **.** **Distribution of non-21 nt vsiRNAs across viral genomes.** (A) Distribution of non-21 nt (between 15 and 29 nt) VSV-derived vsiRNAs (control LacZ-depleted library) across the viral genome. vsiRNAs mapping to genomic strand are depicted in blue, antigenomic strand in red. (B) Distribution of non-21 nt (between 15 and 29 nt) DCV-derived vsiRNAs (control LacZ-depleted library) across the viral genome. vsiRNAs mapping to genomic strand are depicted in blue, antigenomic strand in red. (C) Distribution of non-21 nt (between 15 and 29 nt) VACV-derived vsiRNAs (control GFP-depleted library) across the viral genome. vsiRNAs mapping to the (+) strand are depicted in blue, (−) strand in red. (D) Distribution of non-21 nt (between 15 and 29 nt) RVFV-derived vsiRNAs (control LacZ-depleted library) across the viral genome. vsiRNAs mapping to genomic strand are depicted in blue, antigenomic strand in red.
(TIF)

Figure S2 **Related to** **Figures 1** **,** **3** **,** **5** **, and** **7** **. miRNA and siRNA pathway components are depleted by RNAi.** (A) Robust loss-of-function phenotypes were verified by small RNA northern blotting of RNA samples used to create small RNA libraries. Loss of miRNA pathway components (left panel) affected steady-state levels of bantam miRNA (mature and pre-miRNA forms), while loss of siRNA pathway components resulted in a depletion of the endogenous siRNA esi-2.1. Equal loading was verified by probing 2S rRNA. (B) OligodT-primed cDNA from cells depleted of the indicated RNA silencing factor was subjected to PCR and quantified relative to Rp49 levels. Data is expressed as the normalized mRNA expression in the depleted cells compared to the control cells bathed in a non-targeting dsRNA (LacZ). A representative experiment is shown. Error bars indicate standard deviation.
(TIF)

Figure S3 **Related to** **Figure 2** **. Detection of VSV mRNA and putative DI hairpin RNAs.** Northern blot from Figure 2, probed for various VSV-derived RNA species, which now includes a longer exposure of the rightmost panel in order to better visualize the low-abundance VSV-L mRNA species (∼6 kb), indicated by arrow.
(TIF)

Figure S4 **Related to** **Figure 8** **.** **VACV terminal repeat-derived siRNA biogenesis and Argonaute stabilization.** (A) vsiRNAs produced from a “hot spot” region may be generated due to bidirectional transcription, which generates complementary RNAs with the potential to base pair and form a dsRNA target of the RNAi machinery. A VACV vsiRNA “hot spot” region is highlighted in orange, and the two VACV genes that could potentially produce overlapping, bidirectional transcripts are diagrammed in green. (B) The most abundant repeat-derived vsiRNAs of each size class are diagrammed in reference to the terminal repeat sequence, shown in green. Their relative abundance is calculated as a percentage of all repeat-derived vsiRNAs with >10 reads cloned in the control library. (C) Quantification of the abundant vsiRNAs described in *(A)* upon Dcr-1 and Dcr-2 depletion. (D) Quantification of the abundant vsiRNAs described in *(A)* upon Ago1 and Ago2 depletion.
(TIF)

Table S1 **Related to** **Figure 1** **.** Start location, end location, size, copy number, mismatches and strand for VSV vsiRNA libraries.
(XLSB)

Table S2 **Related to** **Figure 3** **.** Start location, end location, size, copy number, mismatches and strand for RVFV vsiRNA libraries.
(XLSB)

Table S3 **Related to** **Figure 5** **.** Start location, end location, size, copy number, mismatches and strand for Control, Dcr-2, and Ago2 DCV vsiRNA libraries.
(XLSB)

Table S4 **Related to** **Figure 5** **.** Start location, end location, size, copy number, mismatches and strand for Drosha, Dcr-1 and Ago1 DCV vsiRNA libraries.
(XLSB)

Table S5 **Related to** **Figure 7** **.** Start location, end location, size, copy number, mismatches and strand for VACV vsiRNA libraries.
(XLSB)

Text S1 **Supporting experimental procedures.**
(DOCX)
