# Supplementary figures and images for: Dicer-2 Processes Diverse Viral RNA Species
Source: PLoS One. 2013 Feb 12;8(2):e55458. doi: 10.1371/journal.pone.0055458 (PMC3570552; doi:10.1371/journal.pone.0055458)

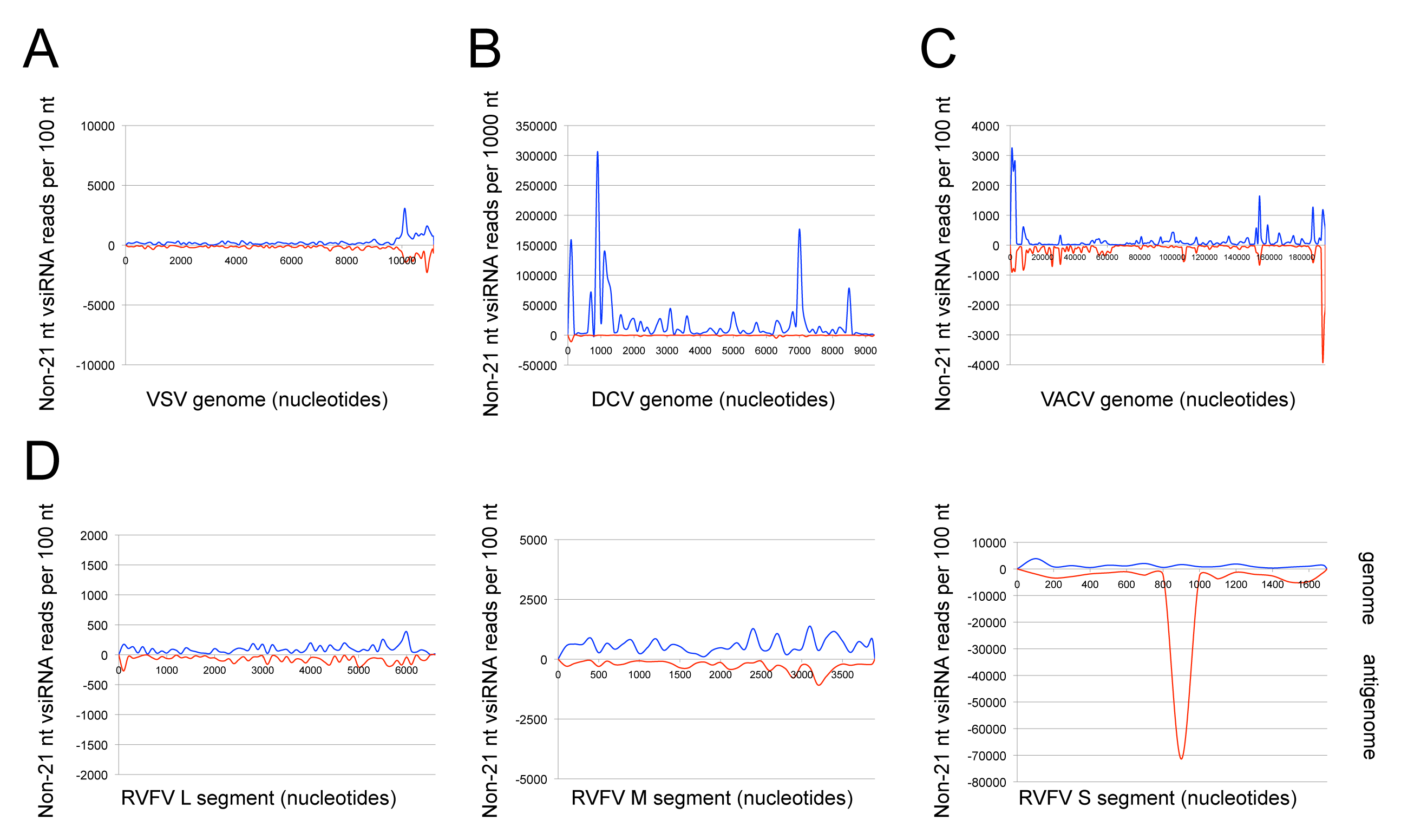

Supplement: Figure S1 — Related to Figures 1 , 3 , 5 , and 7 . Distribution of non-21 nt vsiRNAs across viral genomes. (A) Distribution of non-21 nt (between 15 and 29 nt) VSV-derived vsiRNAs (control LacZ-depleted library) across the viral genome. vsiRNAs mapping to genomic strand are depicted in blue, antigenomic strand in red. (B) Distribution of non-21 nt (between 15 and 29 nt) DCV-derived vsiRNAs (control LacZ-depleted library) across the viral genome. vsiRNAs mapping to genomic strand are depicted in blue, antigenomic strand in red. (C) Distribution of non-21 nt (between 15 and 29 nt) VACV-derived vsiRNAs (control GFP-depleted library) across the viral genome. vsiRNAs mapping to the (+) strand are depicted in blue, (−) strand in red. (D) Distribution of non-21 nt (between 15 and 29 nt) RVFV-derived vsiRNAs (control LacZ-depleted library) across the viral genome. vsiRNAs mapping to genomic strand are depicted in blue, antigenomic strand in red. (TIF) [file pone.0055458.s001.tif]

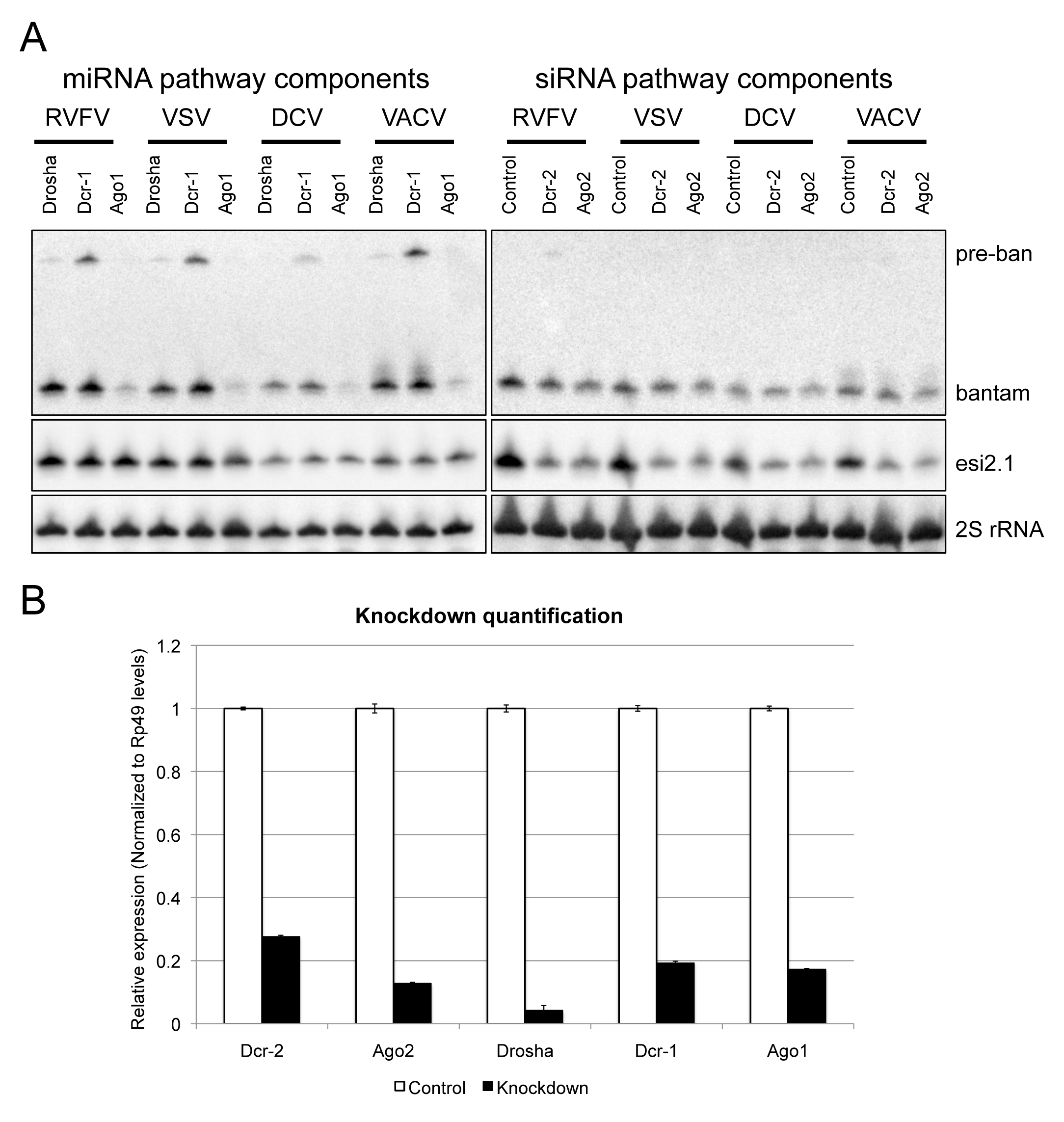

Supplement: Figure S2 — Related to Figures 1 , 3 , 5 , and 7 . miRNA and siRNA pathway components are depleted by RNAi. (A) Robust loss-of-function phenotypes were verified by small RNA northern blotting of RNA samples used to create small RNA libraries. Loss of miRNA pathway components (left panel) affected steady-state levels of bantam miRNA (mature and pre-miRNA forms), while loss of siRNA pathway components resulted in a depletion of the endogenous siRNA esi-2.1. Equal loading was verified by probing 2S rRNA. (B) OligodT-primed cDNA from cells depleted of the indicated RNA silencing factor was subjected to PCR and quantified relative to Rp49 levels. Data is expressed as the normalized mRNA expression in the depleted cells compared to the control cells bathed in a non-targeting dsRNA (LacZ). A representative experiment is shown. Error bars indicate standard deviation. (TIF) [file pone.0055458.s002.tif]

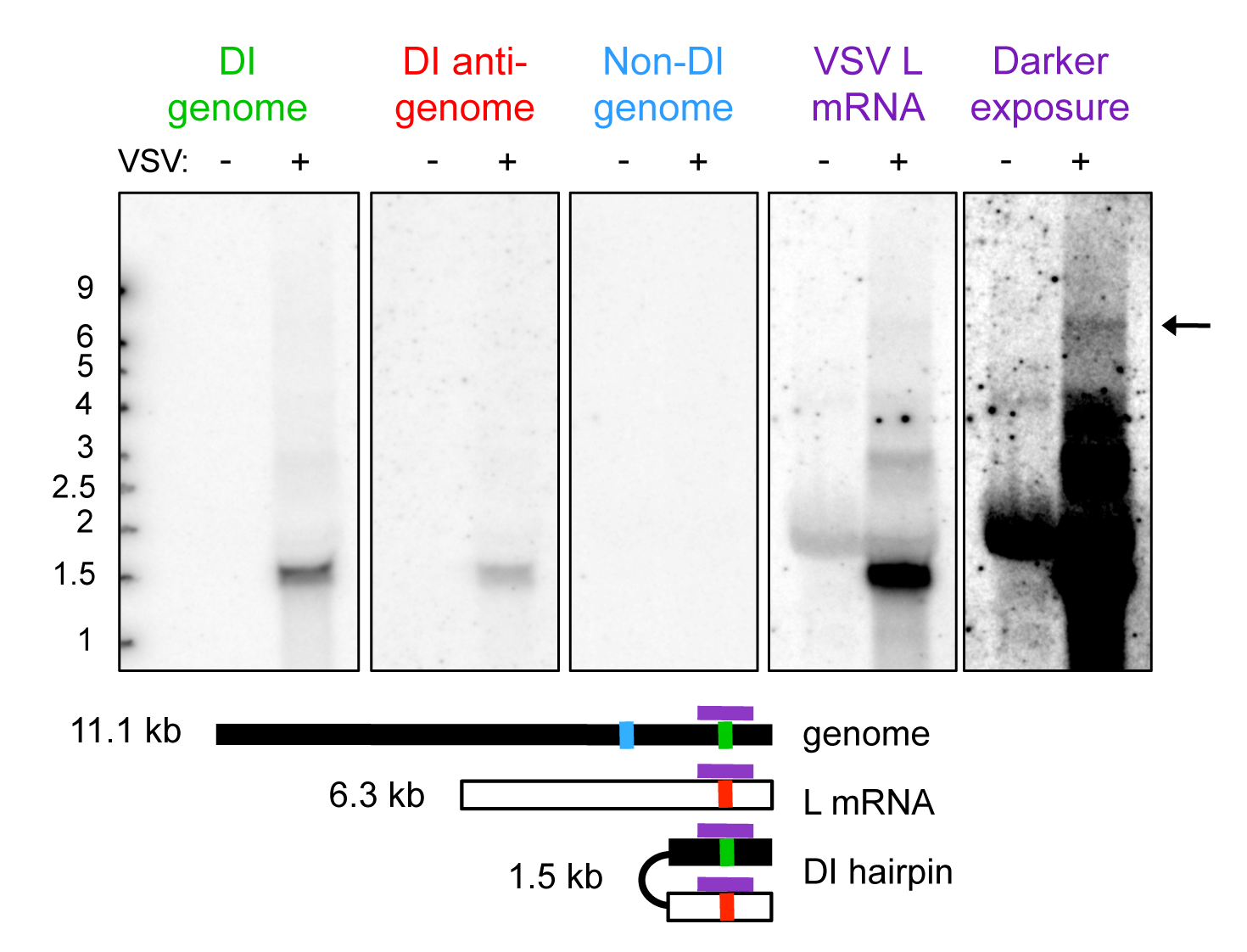

Supplement: Figure S3 — Related to Figure 2 . Detection of VSV mRNA and putative DI hairpin RNAs. Northern blot from Figure 2, probed for various VSV-derived RNA species, which now includes a longer exposure of the rightmost panel in order to better visualize the low-abundance VSV-L mRNA species (∼6 kb), indicated by arrow. (TIF) [file pone.0055458.s003.tif]

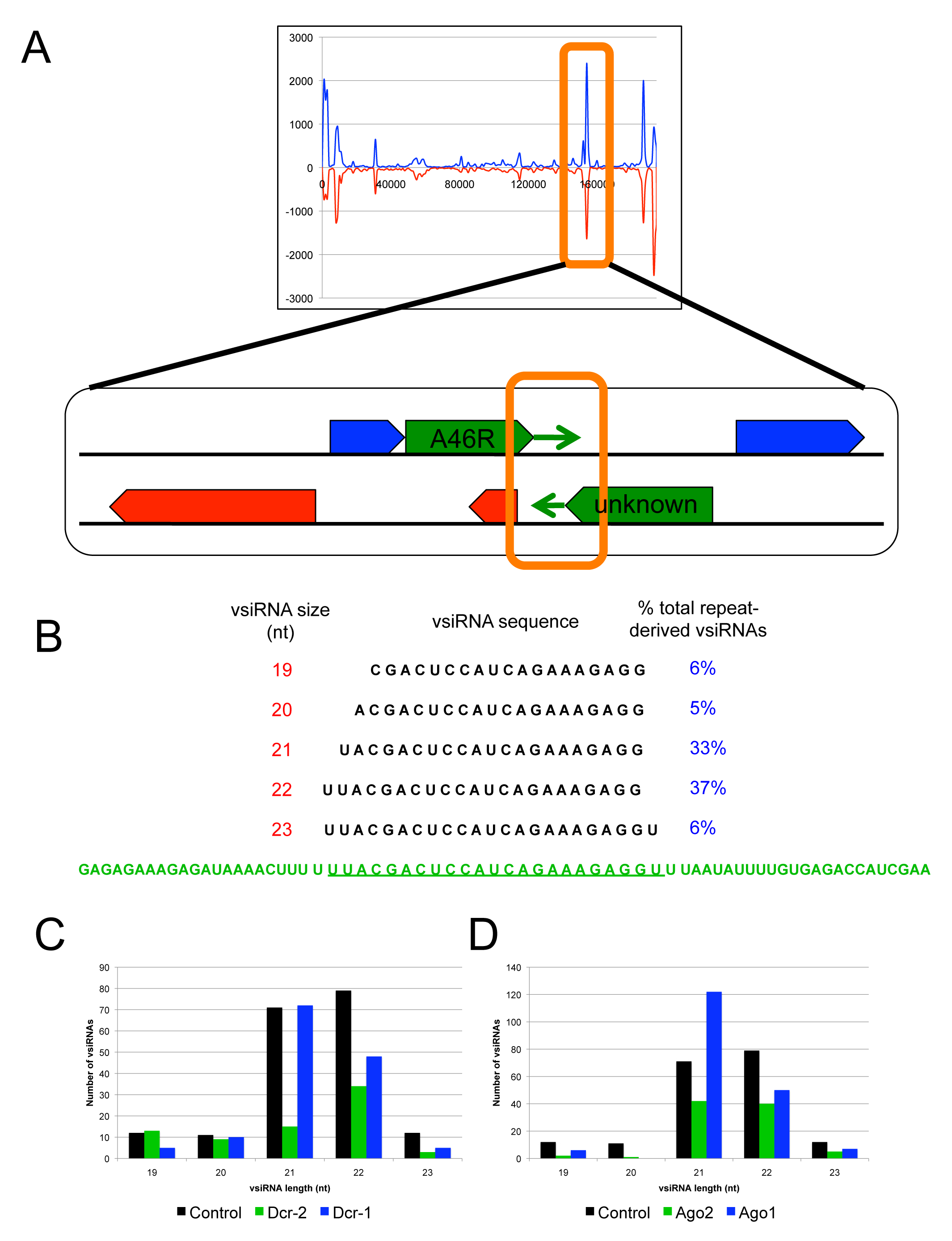

Supplement: Figure S4 — Related to Figure 8 . VACV terminal repeat-derived siRNA biogenesis and Argonaute stabilization. (A) vsiRNAs produced from a “hot spot” region may be generated due to bidirectional transcription, which generates complementary RNAs with the potential to base pair and form a dsRNA target of the RNAi machinery. A VACV vsiRNA “hot spot” region is highlighted in orange, and the two VACV genes that could potentially produce overlapping, bidirectional transcripts are diagrammed in green. (B) The most abundant repeat-derived vsiRNAs of each size class are diagrammed in reference to the terminal repeat sequence, shown in green. Their relative abundance is calculated as a percentage of all repeat-derived vsiRNAs with >10 reads cloned in the control library. (C) Quantification of the abundant vsiRNAs described in (A) upon Dcr-1 and Dcr-2 depletion. (D) Quantification of the abundant vsiRNAs described in (A) upon Ago1 and Ago2 depletion. (TIF) [file pone.0055458.s004.tif]
