# Supplementary material for: Dicer-2 Processes Diverse Viral RNA Species
Source: PLoS One. 2013 Feb 12;8(2):e55458. doi: 10.1371/journal.pone.0055458 (PMC3570552; doi:10.1371/journal.pone.0055458)
Supplement: Text S1 — Supporting experimental procedures. (DOCX) [file pone.0055458.s010.docx]

**SUPPORTING Experimental Procedures**

**Oligo sequences**

RVFV S vsiRNAs: 5’-CCCCCCACCCCCTAATCCCGACCGTAAC-3’

tRNA^val^ : 5’-TGGTGTTTCCGCCCGGGAA-3’

VACV Forward: TCGAAGAGAGAAAGAGATAAAACTT

VACV Reverse: CTTTGGTACAAAATTTCACACAAGT

VSV DI genome [recognizes the - sense genomic strand (nt10500-10550)]: GACCTGCAGATTTATTGACCATTAGCCTTTTTTATATGGCGATTATATCGT
VSV DI antigenome [recognizes the + sense antigenomic strand (nt10500-10550)]: ACGATATAATCGCCATATAAAAAAGGCTAATGGTCAATAAATCTGCAGGTC
VSV non-DI genome [recognizes the - sense genomic strand (nt9300-9350)]: CTATTTCCACCACCCTCTTGCAAATCCTATACAAGCCATTTTTATCTGGGA
VSV-L Forward: TAATACGACTCACTATAGGGGATCCCAATTCATTCCTGATCC

VSV-L Reverse: TAATACGACTCACTATAGGGGATCCCTTTTGAAATTCGTCTA

[VSV-L F/R amplify a 600nt region (VSV nt10400-11000)]

qDroshaF-pub: TCACCATCCACGAGCTAGACAT

qDroshaR-pub: CCTTTCCATTATCTGGCAGGTC

qDicer-1 F: ACGGAGCCAGAAGACACAAGTGAT

qDicer-1 R: GTGCCATTTGATGCACCAAGTCCT

qAgo1 F: ACGCGGATACCAAGAAGGTCATGT

qAgo1 R: GCTTTCATCCGCTTTCCGCTTTCT

qDicer-2 F: TCCACAAACGTGGATGTGCCAAAG

qDicer-2 R: AGCTCCGGCTCGAAGAGATTGAAA

qAgo2 F: ACTACCCATTGAAGTTTCCCC

qAgo2 R: CCTGAGTTGCTCCATCCTTG

**Mapping smRNA reads**

Sequence information was extracted from the image files with the Illumina base calling software package (GAPipeline version 1.4). Prior to alignment of the smRNA reads, ‘‘vectorstrip’’ in the EMBOSS package was used to identify the first eight bases of the 3’ adaptor sequence, and the sequencing read was truncated up to the junction with the adaptor sequence. No further analysis was performed on reads that did not contain adaptor sequence, as those reads lacking an adaptor cannot be precisely sized. The smRNA reads were then reduced to a list of only non-redundant (NR) sequences to minimize the computational requirement in all following procedures. It is of note that the number of times each smRNA was sequenced is maintained during this reduction step. The NR smRNA reads with perfect matches of length 15 – 29 nt were aligned and parsed using in-house Perl scripts to obtain the genomic location of smRNAs on the viral genome (Tables S1-S4).

**vsiRNA profiles**

Viral genomic regions were divided into uniformly sized bins. For VACV, we utilized a bin size of 1000 nt, while the genomes of all other viruses were broken into 100 nt partitions. Finally, absolute vsiRNA content for both the genomic and antigenomic strand of each interrogated virus was determined for each uniformly sized bin. To compare relative vsiRNA abundances between knockdown conditions, absolute vsiRNA values were normalized to the control library based on the total number of mapped reads, then plotted in relation to their genomic position.

**RNA secondary structure predictions**

All structure predictions were performed using the RNAfold software available through the Vienna RNA webservers at http://rna.tbi.univie.ac.at/cgi-bin/RNAfold.cgi.
